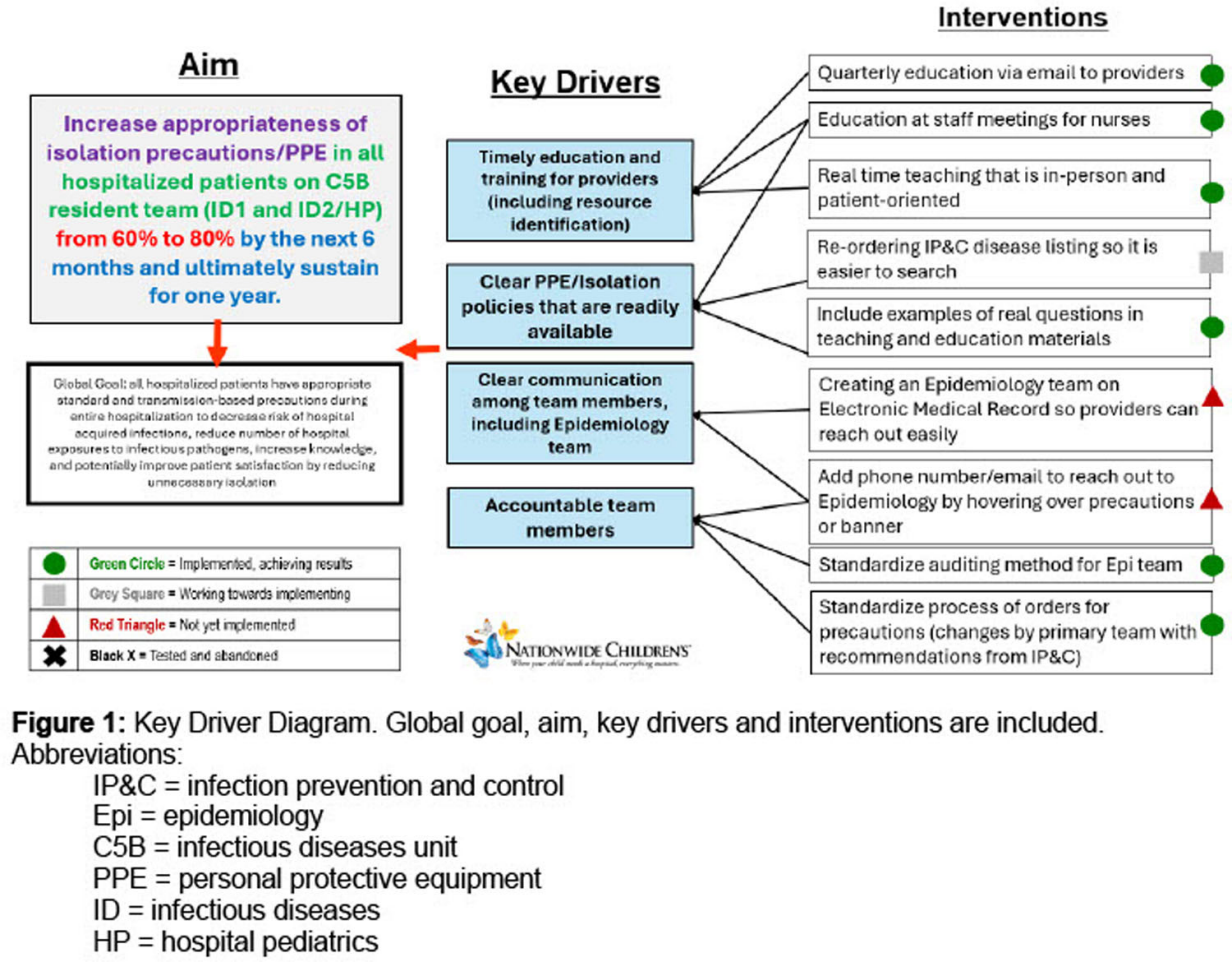# 348 MRSA Colonization Rates Among Healthcare Workers at a Los Angeles County Teaching Hospital

**DOI:** 10.1017/ash.2026.10688

**Published:** 2026-06-23

**Authors:** Samiksha Tarun, Vanessa Yergin, Payal Patel, Sarah King, Matthew Washam

**Affiliations:** 1 Nationwide Children’s Hospital - OSU; 2 Nationwide Children’s Hospital

## Abstract

**Background:** Appropriate implementation of Transmission-Based Precautions (TBP) are necessary to decrease the risk of exposure to infectious pathogens and hospital-acquired infections (HAI). Through routine auditing, gaps were identified in the appropriateness of TBP orders within our institution. A multi-disciplinary quality improvement team was convened to implement a TBP stewardship pilot program with aim to increase TBP compliance and optimize use of personal protective equipment (PPE). **Methods:** Preintervention planning: A key-driver diagram (Figure 1) was created for the pilot program following multiple meetings with infectious diseases (ID) physicians, healthcare epidemiologists, infection preventionists (IP), and nursing leadership. Patients admitted to the ID ward and under the management of the ID service, consisting of 3 pediatrics residents, an ID Pharmacist, and the ID attending physician, were selected for the pilot program starting in October 2025. Protocol Design: Education materials were developed and distributed to members of the ID service, including nursing staff. The stewardship pilot program team, consisting of 4 IPs and 2 healthcare epidemiologists, conducted twice weekly chart reviews of ID ward patients to determine the appropriateness of TBP orders. The pilot program team would interface in-person to review the admitted patients with the ID service once weekly. To determine the impact of the pilot program, baseline and post-intervention TBP-appropriateness was compared using the chi-square test of proportions. **Results:** At baseline, 60% of patients were on appropriate TBP (n = 249). Following the implementation of the pilot program, the percentage of patients on appropriate TBP increased to 73% (n = 162), p < 0.01. During the interim analysis period, there were no noted outbreaks or documented HAIs on the ID ward. **Conclusion:** An interim analysis of the TBP stewardship pilot program demonstrated an improvement in the appropriateness of TBP applied to hospitalized patients. Based on qualitative comments from the physicians and nursing staff, the pilot program was well-received. Strengths of the program include real-time feedback, education, and review as well as increased visibility of the Infection Prevention & Control team. Future efforts will focus on increasing TBP-appropriateness to the 80% goal and quantifying the impact on PPE utilization.

**Figure:**